# Evaluation of the effects of 80% methanolic leaf extract of *Caylusea abyssinica (fresen.)* fisch. & Mey. on glucose handling in normal, glucose loaded and diabetic rodents

**DOI:** 10.1186/1472-6882-12-151

**Published:** 2012-09-11

**Authors:** Wondmagegn Tamiru, Ephrem Engidawork, Kaleab Asres

**Affiliations:** 1 Department of Pharmacology and Therapeutics, School of Pharmacy, Addis Ababa University, P.O, Box 1176, Addis Ababa, Ethiopia; 2 Department of Pharmacognosy and Pharmaceutical Chemistry, School of Pharmacy, Addis Ababa University, Addis Ababa, Ethiopia

**Keywords:** *Caylusea abyssinica*, Diabetes, Hypoglycemic effect

## Abstract

**Background:**

The leaves of *Caylusea abyssinica (fresen.) Fisch. & Mey.* (Resedaceae), a plant widely distributed in East African countries, have been used for management of diabetes mellitus in Ethiopian folklore medicine. However, its use has not been scientifically validated. The present study was undertaken to investigate antidiabetic effects of the hydroalcoholic leaf extract of *C. abyssinica* extract in rodents.

**Materials and method:**

Male Animals were randomly divided into five groups for each diabetic, normoglycemic and oral glucose tolerance test (OGTT) studies. Group 1 served as controls and administered 2% Tween-80 in distilled water, (TW80); Group 2 received 5 mg/kg glibenclamide (GL5); Groups 3, 4 and 5 were given 100 (CA100), 200 (CA200) and 300 (CA300) mg/kg, respectively, of the hydroalcoholic extract of *C. abyssinica.* Blood samples were then collected at different time points to determine blood glucose levels (BGL). Data were analyzed using one way ANOVA followed by Dunnet’s post hoc test and p < 0.05was considered as statistically significant.

**Results:**

In normal mice, CA200 and GL5 induced hypoglycemia starting from the 2^nd^ h but the hypoglycemic effect of CA300 was delayed and appeared at the 4^th^ h (p < 0.05 in all cases). In diabetic mice, BGL was significantly reduced by CA100 (p < 0.05) and CA300 (p < 0.01) starting from the 3^rd^ h, whereas CA200 (p < 0.001) and GL5 (p < 0.05) attained this effect as early as the 2^nd^ h. In OGTT, TW80 (p < 0.01) and CA100 (p < 0.01) brought down BGL significantly at 120 min, while CA200 (p < 0.001) and GL5 (p < 0.001) achieved this effect at 60 min indicating the oral glucose load improving activity of the extract. By contrast, CA300 was observed to have no effect on OGTT. Acute toxicity study revealed the safety of the extract even at a dose of 2000 mg/kg. Preliminary phytochemical study demonstrated the presence of various secondary metabolites, including, among others, saponins, flavonoids and alkaloids.

**Conclusion:**

The results indicate that *C. abyssinica* is endowed with antidiabetic and oral glucose tolerance improving actions, particularly at the dose of 200 mg/kg in experimental animals. These activities of the plant extract may be related to the presence of secondary metabolites implicated in antidiabetic activities of plant extracts via different hepatic and extra-hepatic mechanisms. These results thus support the traditional use of the leaf extract for the management of diabetes mellitus.

## Background

Diabetes mellitus (DM) is a group of metabolic disorders characterized by the presence of a chronic hyperglycemia due to defective insulin secretion and/or insulin action. It is also associated with dysfunctions in carbohydrate, fat and protein metabolism [1,2]. Chronic hyperglycemia can lead to long-term complications and tissue damages such as retinopathy, nephropathy and/or neuropathy, often associated with serious diseases, which are devastating to the individual and very expensive to the health care services [3]. The worldwide prevalence of DM has risen dramatically over the past two decades at alarming rates. The global prevalence for 2010 was 6.4% and predicted to rise to 7.1% by the year 2030 [3,4]. Several treatment strategies are currently used for managing DM and early intervention is needed in order to minimize the risk of macrovascular disease such as cardiovascular disorders [5]. Insulin and oral hypoglycemic agents as well as diet and exercise may be used in the management of DM. In spite of the introduction of hypoglycemic agents, diabetes and the related complications continue to be a major health problem worldwide [6].

Since time immemorial, plant extracts have been used to treat patients with DM in various parts of the world. Currently, especially in developing countries, many plants were listed to be used for the management of diabetes [7-10]. A large number of these plants or their preparation have been evaluated and confirmed to have hypoglycemic effects in animal models [11,12]. Some have also been evaluated in human beings [13-15]. Most of these plants contain glycosides, alkaloids, terpenoids, flavonoids, polysaccharides, and saponins, which are frequently implicated to having anti-diabetic effect [16,17]. However, much is not known about the specific mechanism of action of these plants, although insulinomimetic activity has been proposed for some [18].

The practice of using plants for management of diabetes is also documented in Ethiopia just like other ailments. The leaves of *Caylusea abyssinica (fresen.) Fisch. & Mey.* (family, Resedaceae) have been used in the treatment of DM in Ethiopian folk-medicine without any scientific proof for safety and efficacy [19]. Thus, investigating the safety and efficacy of this plant in animal model could give valuable information to the public at large and also serves as baseline data for researchers engaged in search of medicinal plants with antidiabetic activity. The World Health Organization Expert Committee on diabetes recommended that traditional medicinal herbs be further investigated as they are frequently considered to be less toxic and free from side effects [20]. Therefore, search for safe and more effective agents has continued to be an important area of active research. The present study was therefore undertaken to investigate antidiabetic effects of the 80% methanolic leaf extract of *C. abyssinica* in rodents.

## Method

### Drugs and chemicals

The following drugs and chemicals were used in the experiment: Streptozocin (Chengdu Yuyang High-tech Developing Co.,Ltd, China), glucose standard strip/kits (GLAB, Germany), glibenclamide (Sanofi-Aventis, USA), one touch glucometer (GLAB, Germany), Tween-80 (BDH Laboratory supplies Ltd, England), methanol absolute acetone free (ReAgent Chemical Services Ltd, UK), hydrochloric acid (BDH Ltd, England,) chloroform (ACS, ISO, Merck), sulfuric acid (Farm Italia Carrloerba, Italy), acetic anhydride (Techno Pharmchem, India), ferric chloride (FISHER Scientific Company, New Jersey), potassium ferrocyanide (BDH Ltd, England), ferric sulfate (BDH Ltd, England), lead acetate (BDH Ltd, England), and ethyl acetate (ACS, Merck).

### Plant material

*C. abyssinica (fresen.)* Fisch. & Mey, was collected from Dirre, 55 km away from Addis Ababa (10 km on the roadway from Bishoftu to Ziquala) in November 2010. Taxonomic identification was done and a voucher specimen was deposited (voucher specimen number WT/001) at the National Herbarium, College of Natural Sciences, Addis Ababa University.

### Experimental animals

Healthy Male Swiss albino mice (weighing 20–30 g and age of 8–12 weeks) and Wistar rats (weighing 150–200 g and age of 3 months) were obtained from the animal house of Ethiopian Health and Nutrition Research Institute and School of Pharmacy, Addis Ababa University. Animals were housed in polypropylene cages (6–10 animals per cage), maintained under standard condition (12 h light and 12 h dark cycle; 25-30°C) and allowed free access to pellet diet and water *ad libtum*. After randomized grouping and before initiation of the experiment, animals were acclimatized to the laboratory conditions. All procedures complied with The Guide for the Care and Use of Laboratory Animals [21] and approved by the Institutional Review Board of the School of Pharmacy, Addis Ababa University.

### Extraction

Leaves of the plant material was thoroughly washed with distilled water to remove dirt and soil, and dried under shade and optimal ventilation. The plant material was then pulverized and the powdered plant material was macerated in 80% methanol for 72 h in three successive volumes. The resultant hydro-alcoholic extract was dried under reduced pressure. The dried extract was kept in a refrigerator until use.

### Preliminary phytochemical screening

Standard screening tests of the extract were carried out for various plant constituents. The crude extract was screened for the presence or absence of secondary metabolites such as reducing sugars, alkaloids, steroidal compounds, phenolic compounds, tannins, saponins, flavonoids, cardiac glycosides, and anthraquinones using standard procedures [22,23].

### Acute toxicity test

Acute toxicity test was done based on the limit test recommendations of OECD 425 Guideline [24]. On day one, Swiss albino mouse fasted for 3–4 h was given 2000 mg/kg of the extract orally. The mouse was then kept under strict observation for physical or behavioral changes for 24 h, with special attention during the first 4 h. Following the results from the first mouse, other four mice were recruited and fasted for 3–4 h and administered a single dose of 2000 mg/kg and was observed in the same manner. These observations continued for further 14 days for any signs of overt toxicity.

### Grouping and dosing of animals

Male animals were used for the hypoglycemic and antidiabetic studies based on the results of preliminary study which demonstrated a better percent induction of diabetes by streptozotocin in males than females. Besides, published reports indicate streptozotocin induces severe diabetes in females, with diminished survival rates and they are also less sensitive to insulin compared to male animals [25]. For oral glucose tolerance tests (OGTT), rats were used since they are preferable in such studies [26]. In all cases, group I received 2% Tween-80 in distilled water (TW80) and served as controls; Group II received a standard, 5 mg/kg of glibenclamide, (GL5); Group III-V received 100 mg/kg (CA 100), 200 mg/kg (CA 200) and 300 mg/kg (CA 300). of *C. abyssinica* extract, respectively.

The doses were selected based on the acute toxicity study. The middle dose was one tenth of the limit dose which was 200 mg/kg. Higher dose was calculated as twice the middle dose, which should have been 400 mg/kg. However; the data from preliminary study revealed that 400 mg/kg tended to raise blood glucose level (BGL) and CA 300 was thus taken as a higher dose level. The lower dose level was calculated by taking half of the middle dose, i.e. 100 mg/kg. Volume administered was determined based on OECD guideline that states 2 mL/100 g of body weight of the animal [24]. For the positive control, 5 mg/kg was selected based on earlier reports [27,28]. As people traditionally use the preparations of the plant extract via oral route, the study was conducted using oral route of administration [19].

### Hypoglycemic test in normal mice

Mice were fasted for 4–6 h, but water was allowed *ad libtum,* and then randomly divided into five different groups (6 animals per group). The animals were treated according to their respective grouping. Using aseptic conditions, blood sample was then collected from tail veins of each animal to determine BGL at 0, 1, 2, 3 and 4 h post-treatment. BGL was determined using GLAB strips and GLAB one-touch glucometer. Measurement of BGL was done in triplicate and the average value was taken.

### Assessing antidiabetic activity

Diabetes was induced using streptozocin. The drug was dissolved in 0.1 M citrate buffer (pH = 4.5). The solution was then administered intraperitonially at 150 mg/kg dose to mice that were fasted for 4–6 h prior to administration. Seventy-two hours later, animals were screened for diabetes. Mice which showed fasting BGL > 200 mg/dL were included in the study [29]. Diabetic mice were kept for overnight, each group in a separate cage and were fasted for 4–6 h. The animals were then randomly divided into five groups (n = 9/group) and treated with the extract according to their respective group. Blood samples were collected from the tails of the animals to determine BGL at 0, 1, 2, 3 and 4 h post-treatment.

### Oral glucose tolerance test

Rats were fasted overnight for 12–14 h and assigned randomly into 5 groups (n = 6/group), each group in separate cage and baseline BGL was determined. Thirty minutes before extract treatment, all of the rats were loaded with 2 g/kg glucose solution and then orally treated according to their respective grouping. Blood samples were then collected to determine BGL prior to treatment and after 30, 60 and 120 min of treatment as described above.

### Statistical analysis

All data were expressed as mean ± SEM and percent changes. Between and within group analysis was carried out using one way ANOVA followed by Dunnet’s post hoc test and level of significance was set at p < 0.05. For data processing, SPSS data analysis software Version 19.0 was used.

## Results

### Extraction

The percentage yield of 80% methanolic extract of the dried leaves of *C. abyssinica* was found to be 18.5% (w/w). The extract was dark-brown semisolid at room temperature and solidified when stored in a refrigerator. Extract returned to semisolid state on re-exposure to room temperature.

### Preliminary phytochemical screening

Phytochemical screening of the crude extract of *C. abyssinica* revealed the presence of various secondary metabolites (Table 1). Alkaloids, cardiac glycosides, reducing sugars, steroidal compounds and phenolic compounds, tannins, saponins and flavonoids were detected in the crude extract.

**Table 1:** **Phytochemical screening results of the 80% methanolic extract of the leaves of*Caylusea abyssinica***

**TEST**	**RESULT**
Alkaloids	**+**
Tannins	**+**
Saponins	**+**
Anthraquinone glycosides	**-**
O-anthraquinones	**-**
Cardiac glycosides	**+**
Flavonoids	**+**
Reducing sugars	**+**
Phlabotannins	**-**
Steroidal compounds	**+**
Phenolic compounds	**+**

**+** : present, **–** : absent.

### Acute toxicity study

Acute toxicity study of the hydroalcoholic extract of *C. abyssinica* did not reveal any behavioral, neurological, autonomic or physical changes such as alertness, motor activity, restlessness, convulsions, coma, diarrhea and lacrimation. Besides, the extract did not cause mortality in the animals at a dose of 2000 mg/kg during the observation time. Thus, the median lethal dose (LD50) of the plant extract is said to be greater than 2000 mg/kg, indicating a good safety margin.

### Effects on normoglycemic mice

Results of the effect of *C. abyssinica* on BGL of normal mice are presented in Table 2. Within group analysis revealed that TW80 treated animals did not show significant reduction in BGL across all time points compared to the fasting (initial or baseline) level, although percent reduction tended to be higher at the 3^rd^ (30.9%) and 4^th^ (23.8%) h. Similarly, CA100 failed to show significant hypoglycemic effect at all-time points, though percent reduction appeared to be higher at the 3^rd^ (42.5%) and 4^th^ (42.1%) h. By contrast, CA200 produced a significant (p < 0.05) reduction in BGL at the 2^nd^ (47%), 3^rd^ (49.4%) and 4^th^ (44%) h post treatment compared to the initial level. Likewise, GL5 brought about significant reduction at the 2^nd^ (p < 0.05), 3^rd^ (p < 0.01) and 4^th^ h (p < 0.05), by about 37.3%, 42.8% and 29.9%, respectively. However, CA300 was noted to produce significant reduction (p < 0.05, 53.5%) of BGL only at the 4^th^ h.

**Table 2:** **Hypoglycemic effects of 80% methanolic leaf extract of*Caylusea abyssinica*on blood glucose levels in normal mice**

	**Blood glucose level in mg/dL**				
**Group**	**0 h**	**1 h**	**2 h**	**3 h**	**4 h**
TW80	72.00 ± 9.77	66.00 ± 6.51	81.67 ± 10.83	50.33 ± 1.67	54.83 ± 2.97
GL5	85.33 ± 11.61	68.83 ± 9.22	53.5 ± 5.47^**a1,b1**^	48.83 ± 2.44^**a2**^	56.83 ± 6.2^**a1**^
CA100	79.17 ± 25.33	53.5 ± 3.82	58.67 ± 7.42	45.5 ± 3.21	45.83 ± 4.43
CA200	83.33 ± 16.76	54.17 ± 8.53	44.33 ± 6.38^**a1,b1**^	42.17 ± 3.4^**a1**^	46.83 ± 3.05^**a1**^
CA300	96.33 ± 21.44	75.00 ± 16.21	57.17 ± 5.91	50.5 ± 7.11	44.83 ± 3.12^**a1**^

n = 6; CA100 = *C. abyssinica* extract 100 mg/kg, CA200 = *C. abyssinica* extract 200 mg/kg, CA300 = *C. abyssinica* extract 300 mg/kg, TW80 = 2%Tween-80, GL5 = Glibenclamide 5 mg/kg; ^a^ compared with fasting blood glucose level (t = 0 h), ^b^ compared with negative control group; ^1^p < 0.05, ^2^ p < 0.01.

Between groups analysis, on the other hand, did not produce any significant difference in fasting BGL across groups. Significant hypoglycemia was, however, recorded for CA200 and GL5 at the 2^nd^ h (p < 0.05 in both cases) when the groups were compared with TW80. Interestingly, no apparent difference was noted when the different doses of the extract were compared with each other as well as with the positive control at all-time points.

### Effects on streptozocin-induced diabetic mice

Seventy five mice were injected streptoztocin and 50 of them found to be diabetic, with a success rate of 66.7%. Out of the fifty mice, two died before the start of administration of the extract and all the rest survived until the end of the experiment. The effects of *C. abyssinica* on streptozotocin-induced diabetes are shown in Table 3. Intra-group analysis demonstrated that TW80 had no effect on BGL at all-time points compared to the initial level. By contrast, treatment with the extract and glibenclamide did produce alterations in BGL compared to initial values. Accordingly, whilst CA100 (p < 0.05 in both cases) and CA300 (p < 0.01 in both cases) produced a significant reduction at the 3^rd^ and 4^th^ h; CA200 (p < 0.001 in all instances) and GL5 (p < 0.05, p < 0.01, and p < 0.001, for the time points, respectively) did show a significant reduction at the 2^nd^, 3^rd^ and 4^th^ h. Maximum reduction was attained at the 4^th^ h, with percent reduction for CA100, C200, C300, GL5 being 52.2%, 62.3%, 52.8%, and 63%, respectively.

**Table 3:** **Antidiabetic effects of 80% methanolic leaf extract of*Caylusea abyssinica*on blood glucose levels in streptozotocin induced mice**

	**Blood glucose level in mg/dL**				
**Group**	**0 h**	**1 h**	**2 h**	**3 h**	**4 h**
TW80	376.56 ± 39.02	432. 78 ± 33.87	338.33 ± 24.22	331.45 ± 47.53	272.00 ± 38.58
GL5	342.44 ± 36.97	306.00 ± 40.97	223.78 ± 31.27^a1, b1^	161.22 ± 27.69^a2, b2^	126.56 ± 22.64^a 3, b2^
CA100	335.33 ± 48.12	269.56 ± 43.34^b1^	215.11 ± 37.27^b1^	179.89 ± 39.39^a1, b1^	160.33 ± 36.98^a1, b1^
CA200	326.44 ± 43.78	268.33 ± 18.28^b1^	167.89 ± 18.01^a3, b2^	147. 78 ± 19.61^a3, b2^	123.00 ± 15.52 ^a3, b2^
CA300	352.11 ± 38.52	318.556 ± 40.49	229.22 ± 30.78^b1^	187.44 ± 36.39 ^a2, b1^	166.22 ± 34.29^a2^

n = 9-10; CA100 = *C. abyssinica* extract 100 mg/kg, CA200 = *C. abyssinica* extract 200 mg/kg, CA300 = *C. abyssinica* extract 300 mg/kg, TW80 = 2%Tween-80, GL5 = Glibenclamide 5 mg/kg; ^a^ compared with fasting blood glucose level (t = 0 h), ^b^ compared with negative control group; ^1^p < 0.05, ^2^ p < 0.01, and ^3^p < 0.001.

In inter-group analysis, no detectable changes were noted between the fasting BGL of all groups. Subsequent analysis showed that CA100 (p < 0.05 in all cases) and CA200 (p < 0.05 at the 1^st^ h and p < 0.01 for the rest) significantly decreased BGL at all-time points compared to TW80 animals. Likewise, GL5 produced a similar pattern, with p-values becoming very significant (p < 0.01 or p < 0.001) from the 2^nd^ h onwards. On the other hand, CA300 was capable of reducing BGL significantly (p < 0.05) only at the 2^nd^ and 3^rd^ h (p < 0.05 in both cases). No detectable changes in BGL were observed either amongst the extract or when extracts were compared with the positive control.

### Effect on oral glucose tolerance test in rats

Effects of the extract of *C. abyssinica* on OGTT are shown in Table 4. BGL of all groups prior to extract administration (0 min) showed no apparent difference compared to each other. All groups, however, showed significant increase by 20-40% (p < 0.05 or p < 0.01) in BGL 30 min following extract administration (one hour after oral glucose loading), confirming the induction of hyperglycemia. Hyperglycemia with glucose challenge was not significantly brought down with TW80 at 60 min but significant difference was achieved at 120 min (p < 0.01, 35.6%). Although CA100 failed to produce detectable changes at 60 min, it brought down glucose level by 45.4% (p < 0.01) at 120 min compared to peak hyperglycemia. CA200, however, significantly improved oral glucose loading at 60 (p < 0.001, 31.8%) and 120 min (p < 0.001, 37.4%), respectively. Likewise, GL5 produced significant improvement of hyperglycemia at 60 (p < 0.001, 40.6%) and 120 min (p < 0.001, 42%). By contrast, CA300 failed to produce significant improvement in BGL following glucose challenge at all-time points.

**Table 4:** **The effects of 80% methanolic leaf extract of*Caylusea abyssinica*on Oral Glucose Tolerance Test in rats**

	**Blood glucose level in mg/dl**			
**Group**	**0 min**	**30 min**	**60 min**	**120 min**
TW80	102.33 ± 3.20	140.50 ± 16.76^a1^	126.83 ± 6.55^d3^	90.50 ± 1.96 ^b2^
GL5	101.83 ± 6.29	125.17 ± 11.47 ^a2^	74.33 ± 8.72^b3,c3^	72.67 ± 8.79^b3^
CA100	116.83 ± 4.19	158.67 ± 15.34 ^a2^	145.83 ± 7.03^d3^	86.67 ± 5.82^b3^
CA200	104.00 ± 1.98	131.50 ± 8.68^a1^	89.67 ± 4.90^b3,c2^	82.33 ± 4.45^b3,e2^
CA300	103.50 ± 9.21	127.17 ± 13.34^a1^	117.33 ± 9.06^d3^	100.00 ± 12.03

n = 6; CA100 = *C. abyssinica* extract 100 mg/kg, CA200 = *C. abyssinica* extract 200 mg/kg, CA300 = *C. abyssinica* extract 300 mg/kg, TW80 = 2%Tween-80, GL5 = Glibenclamide 5 mg/kg; ^a^ compared to blood glucose levels (BGL) at 0 min, ^b^ compared with BGL after 30 min, ^c^ compared with the negative control, ^d^ compared with the positive control; ^1^p < 0.05, ^2^ p < 0.01 and ^3^ p < 0.001. Time refers to time after extract administration.

Inter-group analysis, on the other hand, showed that GL5 and CA 200 were capable of reducing BGL significantly (p < 0.001and p < 0.01, respectively) at 60 min compared with TW80. However, BGL was not significantly different between GL5, CA200 and TW80 at 120 min. None of the three doses showed significant difference amongst each other at all-time points. In contrast, GL5 treated groups exhibited significant improvement (p < 0.001) at 60 min compared to CA100.

## Discussion

Developing agents for management of DM that are devoid of adverse effects are still a challenge to the medical care system. Thus, research is increasingly done on medicinal plants with the hope of developing a relatively safe antidiabetic plant-based product alone or in combination with other agents [29]. In this study, no detectable changes were observed in baseline BGL across groups in both normal (Table 2) as well as diabetic animals (Table 3), however, significant reductions started to appear when the hydroalcoholic extract and the standard drug were administered, indicating that changes produced were attributed to treatments received. Therefore, the results of this study indicated that hydroalcoholic extract of *C. abyssinica* reduces BGL level in normal and diabetic mice as well as in glucose induced hyperglycemic rats.

Among the various doses of the extract, maximum activity was observed with CA200 in all tests. It is interesting to note that CA200 was capable of bringing down streptozocin-induced hyperglycemia close to TW80 and GL-5 values (Table 3). The extract also brought the hyperglycemic state in OGTT down within 60 min in the same manner to that of glybenclamide (Table 4). Thus, it is plausible to assume that the plant extract and glibenclamide might produce hypoglycemic and antidiabetic effects by a similar mechanism (i.e. by enhancing insulin release or insulin-like effect). Extracts of other plants, including *Cassia italic*[30] and *Vinca rosea*[27] have been reported to have similar mode of action with that of glibenclamide, lending evidence to this assertion.

It was observed that the extract exerted its action in a non-dose dependent manner, particularly the higher dose produced less activity. CA300 did produce a delayed but significant hypoglycemia and antidiabetic action, although it did not improve oral glucose tolerance. The highest dose determined was 400 mg/kg, however, this dose produced an increase in BGL during the preliminary analysis that led to its exclusion from the study. This observation suggests that activity might decrease with dose. BGL reduction, probably, is the net effect of the interplay between various constituents of the extract. It is likely that higher doses may activate non-specifically both BGL lowering and rising mechanisms. Indeed, it has been reported that the presence of interfering substances in plant extracts may diminish hypoglycemic effect [12,31-35]. It is also likely that bigger doses could cause some toxic effect [35] to specific targets of glucose lowering mechanisms, which could have been the target for hypoglycemic agents.

On the other hand, the lower dose (100 mg/kg) of the extract appeared to be ineffective in reducing BGL in normal (Table 2) and glucose loaded animals (Table 4). This could be attributed to inability of the dose to overcome counter-regulatory physiological mechanisms [36], lesser concentration of the active principles to induce hypoglycemia or the small sample size employed that precluded statistical significance. On the other hand, CA100 produced antidiabetic action in streptozocin-induced mice, which might imply that the hypoglycemic nature of the lower dose would be apparent when there is an alteration in normal blood glucose regulatory systems by diabetes.

The results also showed that antidiabetic activity of the extract increased with time, as maximal effect was achieved at the 4^th^ h (Table 3). This could mean that the active principles in the extract need time to reach sufficient concentration at the target site, as a similar pattern was observed with other plants displaying anti-diabetic activity [37]. The plant extract showed relatively faster antidiabetic onset of action than the standard drug. However, the doses of *C. abyssinica* extract exhibited a varied onset of action, which probably resulted from interference of other principles, particularly at higher doses. Constituents such as reducing sugars that have a higher glycemic index could give rise to free glucose after digestion and they may tend to raise BGL following absorption. The appearance of such an effect in the face of the hypoglycemic actions by the active agents could lead to a delay in appearance of the action, especially at higher doses where the extract may result in higher concentrations of such molecules.

Interestingly, in OGTT the hydroalcolic extract showed significant reduction in BGL from 60 min onwards except for the higher dose. This suggests that the extract is endowed with the ability to enhance glucose regulatory mechanisms, reflecting a potential advantage of the extract in minimizing hyperglycemia related complications of diabetes. This is true provided that the extract happens to demonstrate a similar action with repeated administration.

Preliminary phytochemical screening of the hydroalcoholic extract of *C. abyssinica* demonstrated the presence of secondary metabolites (Table 1), which are known to produce hypoglycemic effects in other plants by various mechanisms [18,38,39]. No previous phytochemical reports could be found in the literature concerning the genus Caylusea. However, other members of the family Resedaceae such as *Reseda muricata* and *Randonia africana* have been reported to contain flavonoids, phenolic compounds, glycosides and alkaloids [40], which are in line with the current findings. Thus, the antidiabetic, hypoglycemic and enhanced glucose utilization effect of the hydroalcoholic extract of *C. abyssinica* may be associated with the presence of these different secondary metabolites that act individually or synergistically.

## Conclusion

Taken together, the present study demonstrated that the 80% methanolic extract of *C. abyssinica* exhibited a significant antidiabetic effect in rodents, providing evidence for the traditional claim. The effective dose of the extract was found to be 200 mg/kg, although this dose was associated with the risk of hypoglycemia. Besides, the plant was found to have a greater safety margin, which is coupled with its activity making it a potential herb to develop plant-based products after further investigation.

## Competing interests

The authors declare that they have no competing interests.

## Authors’ contributions

All authors involved in the design and write up of the study, and WT -conducted the actual study and the statistical analysis. All authors approved the submitted version of the manuscript.

## Pre-publication history

The pre-publication history for this paper can be accessed here:

http://www.biomedcentral.com/1472-6882/12/151/prepub
